# Reaction Sintering of Machinable TiB_2_-BN-C Ceramics with In-Situ Formed h-BN Nanostructure

**DOI:** 10.3390/nano12081379

**Published:** 2022-04-18

**Authors:** Oleksii Popov, Dmitry V. Shtansky, Vladimir Vishnyakov, Oleksandra Klepko, Sergey Polishchuk, Magzhan K. Kutzhanov, Elizaveta S. Permyakova, Petro Teselko

**Affiliations:** 1 Faculty of Physics, Taras Shevchenko National University of Kiev, 01033 Kyiv, Ukraine; esko_@ukr.net; 2 Science and Research Centre “Synthesis”, 02139 Kiev, Ukraine; 3 Research Laboratory of Inorganic Nanomaterials, National University of Science and Technology “MISiS”, Leninsky Prospect 4, 119049 Moscow, Russia; shtansky@shs.misis.ru (D.V.S.); kutzhanov.m@misis.ru (M.K.K.); permyakova.es@misis.ru (E.S.P.); 4 Centre for Engineering Materials, University of Huddersfield, Huddersfield HD1 3DH, UK; v.vishnyakov@hud.ac.uk; 5 Inorganic Materials Vapor Phase Department, E. O. Paton Electric Welding Institute, 03150 Kyiv, Ukraine; klepko21@i.ua; 6 Department of Physics of Dispersed Systems, G. V. Kurdyumov Institute for Metal Physics of the N. A. S. of Ukraine, 03142 Kyiv, Ukraine; serg.polis7@gmail.com

**Keywords:** reactive sintering, thermal shock resistance, hetero-modulus ceramics, titanium diboride, boron nitride nanosheets, pseudoplasticity, machinable ceramic

## Abstract

Soft TiB_2_-BN-C hetero-modulus ceramics were sintered with the assistance of in-situ reactions during the hot pressing of TiN-B_4_C precursors. TiB_2_ formation was observed already after the hot pressing at 1100 °C, remaining the only phase identifiable by XRD even after sintering at 1500 °C. Analysis of reaction kinetics allows us to assume that the most probable reaction controlling stage is boron atoms sublimation and gas phase transfer from B_4_C to TiN. Reactive sintering route allows almost full densification of TiB_2_-BN-C composite ceramics at 1900 °C. The processes enable the formation of multilayer h-BN nanosheets inside the TiB_2_ matrix. The manufactured TiB_2_-33BN-13C ceramic with K_1C_ = 5.3 MPa·m^1/2^ and H_V_ = 1.6 GPa is extremely thermal shock-resistant at least up to quenching temperature differential of 800 °C. The sintered UHTC composite can be machined into complex geometry components.

## 1. Introduction

The covalent nature of the interatomic bonds of metal oxides, nitrides, carbides, and borides not only results in the high melting point of ceramics and oxidation resistance but also shifts the strength loss homologous temperature (the ratio of the current temperature to the material melting point) from 0.2 ÷ 0.3 for metals to 0.5 ÷ 0.7. This makes ceramics a key candidate for high-temperature applications [1,2,3,4,5], despite well-known drawbacks of susceptibility to sharp temperature variations, sintering challenges, and shaping/forming difficulties [6,7].

Hetero-modulus ceramics (HMCs), being a composition of a super hard matrix with soft graphite or h-BN inclusions, demonstrate essential improvements in brittle material thermal shock resistance. It has also been shown that the materials are machinable and the inclusions have no negative impact on their high-temperature properties. Everything mentioned facilitates the industrial use of HMCs [8,9,10,11,12,13,14,15,16]. As is shown in several works, both thermal shock resistance and machinability raise significantly after adding 20 vol.% of the soft phase [9,12,14,17,18]. However, poor sinterability of the graphite and h-BN hinders the material densification and leads to the need for higher sintering parameters [19,20]. E.g., to obtain non-porous TiC with 30 vol.% of C ceramic, Shabalin et al. [15] hot pressed the green body at 2700 °C and 12 MPa. The application of the same parameters for TiC ceramic with 40 vol.% of C resulted in 5% porosity. A similar sintering route has been used by John and Jenkins for C-MeC composites [21]. TiB_2_ with 50 vol.% of hBN ceramics had been hot-pressed at 1900 °C and 30 MPa for 60 min by Akarsu et al., but the resulting material contained approximately 30% of pores [22]. Reduced porosity in BN-based material was obtained with the addition of B_2_O_3_ as a fusible sintering aid when ceramic has been densified up to 92% at 1900 °C and 50 MPa for 60 min [23]. Bearing in mind that TiB_2_-BN based ceramics are being widely used for aluminum evaporation boats [24] and as a side-dam material for thin-strip casting in steel production [23], the optimization of the high-quality HMC sintering procedure remains an important task.

The reaction sintering technique, which is when all material phases are formed in-situ via the high-temperature chemical interaction between the precursor powders, was shown to intensify material consolidation and lower porosity and allowed for less demanding sintering conditions [25,26,27,28,29,30,31]. In addition, the reaction hot pressing of hetero-modulus ceramics allowed the production of submicron graphite or even graphene platelets [32]. The platelets efficiently blunt a crack tip and by this process increase the ceramic toughness [11,33]. TiB_2_-C based ceramics were efficiently manufactured by means of the hot pressing of TiC-B_4_C precursors (e.g., green bodies) by utilizing the following reaction:

2MeC + B_4_C → 2MeB_2_ + 3C (1)

The non-porous UHTCs were produced at 1800–1900 °C during 2–16 min of pressing [11,34]. It was shown that the reactively formed submicron graphite platelets improved the ceramic matrix thermal shock resistance. However, the content of the soft phase, which one can obtain in the titanium diboride matrix by utilizing reaction (1), cannot exceed 30 vol.%. Zou et al., showed that the hot pressing of ZrN, B_4_C and Si powder mixture resulted in ZrB_2_-BN-SiC composite with an h-BN content of 28 vol.%. The h-BN was homogeneously distributed in the ZrB_2_-SiC matrix [26]. It is evident that a similar process with no silicon in the green body should prevent the carbon from forming hard SiC crystals and therefore increase the soft phase content. The presented work is the investigation of the possibility to create soft TiB_2_-BN-C hetero-modulus ceramics using the reaction of the hot pressing of TiN-B_4_C precursors. The soft phase content increasing should significantly improve the UHT material machinability and thermal shock resistance with no negative impact on high temperature properties. The reactive sintering approach would simplify the ceramic processing.

## 2. Experimental Procedure

### 2.1. Sample Sintering and Preparation

Commercially available powders of TiN (~30 μm) and B_4_C (~20 μm) (Donetsk Reactive Factory, Donetsk, Ukraine) were used as green body materials. The materials purity, as stated by the manufacturers, was around 99.0 at%. The green body composition was chosen to comply with the stoichiometry of the following reaction:

4TiN + 3B_4_C → 4TiB_2_ + 4BN + 3C (2)

Two green body mixtures entitled “reactive” (4TiN + 3B_4_C) and “nonreactive” (4TiB_2_ + 4BN + 3C) were prepared according to the reaction’s (2) left and right-hand side, respectively (See Table 1).

The powder mixtures were ground in a high-energy planetary mill (1500 r.p.m.) (Physics Faculty, Taras Shevchenko National University of Kyiv, Kyiv, Ukraine) in a zirconia jar with 10 mm zirconia balls for 30 min and hot-pressed at a pressure of 30 MPa in a graphite die, without a special protective atmosphere. The die assembly was heated with an AC (50 Hz) current in the air with the use of the hot-pressing equipment DCS-0 produced by SRC Synthesis (Kiev, Ukraine). The heating rate (after preheating to 600 °C for 20 min) was approximately 100°/min. The synthesis temperature for the reactive powder ranged from 1000 to 1900 °C. The nonreactive powder has been hot pressed at 1900 °C only. The sample thickness has been calculated from the plunger movement, which has been measured in micrometres. The micrometre accuracy was about 10 μm.

### 2.2. Material Characterization

The microstructure of the TiB_2_-BN-C composites was studied on a JSM F7600 (JEOL, Japan) and Tescan Vega3 scanning electron microscope (Tescan, Czech Republic) both equipped with energy-dispersive X-ray (EDX) spectrometers. Raman spectrum was recorded on a Thermo DXR spectrometer (Thermo Fisher Scientific, Waltham, WI, USA) with a 532 nm excitation laser.

The bulk densities of obtained materials were measured using Archimedes’ method. The relative densities were calculated based on the theoretical densities of TiB_2_ (4.495 g/cm^3^), BN (2.1 g/cm^3^), and C (graphite) (2.28 g/cm^3^) assuming the rule of mixtures. X-ray diffraction (DRON-4M) with Cu-Kα radiation analyzed with Rietveld method according to the procedure presented in [35] was used to identify the phase composition of the specimens. Chemical composition was determined by Energy Dispersive X-ray Spectroscopy (EDS) (Oxford, UK) analysis.

Indentation measurements (hardness and fracture toughness) were performed with a load of 98 N and a 10 s holding at the load. The measurements were taken on the polished sample surfaces. The fracture toughness was estimated by measuring the crack lengths generated by the Vickers indentations. The toughness was calculated according to the formula of Niihara et al. [36].

Thermal shock crack growth was examined according to the indentation-quench method, as described elsewhere [37]. The disk specimens were polished and indented using a Vickers pyramidal indenter as described above. Five indentations were made on each specimen. Four cracks at the places of pyramid ribs were formed after each indentation. This way, 20 cracks per sample were measured. The samples were heated to a preselected set of temperatures: 200, 300, 400, 500, 600, 700, and 800 °C in the air for 10 min and then quenched in a water bath at room temperature (20 °C). The radial crack lengths of each indentation were measured before and after each quenching event by an optical microscope. The crack growth was expressed as a percentage of the initial crack length as:

(3) $$\frac{\left(c_{i}-c_{0}\right)}{c_{0}}\times 100\%$$

with *i* being the sequential number of the quenching cycle. The average crack growth for each sample was estimated as a function of the quenching temperature.

Room-temperature compressive strength was determined using an AG-Xplus universal tensile testing machine (Shimadzu, Japan). The load increase rate was 8.0 × 10^−5^ m/s.

To reveal the influence of *h*-BN on the material resistance to shock-dynamic loads, the TiB_2_-BN-C and TiB_2_-C composites were subjected to cyclic impact test at an applied load of 300 N using an impact tester (CemeCon) (CemeCon, Germany). Each sample was exposed to 10^5^ impacts with a frequency of 50 Hz using a WC-Co ball with a diameter of 5 mm. The shape and morphology of impact cavities were observed with an NT1100 optical profiler (WYKO).

The degree of conversion α in chemical reactions was estimated based on amounts of titanium in the green body and in the product phases based on the XRD data. If at time *t,* an amount of Ti consisted in the product phase (TiB_2_) was *N_Ti_*(*t*) and the total amount of Ti in both product (TiB_2_) and precursor (TiN) phases was *N_Ti_*_0_ then α was estimated as:

(4) $$\alpha =\frac{N_{Ti}\left(t\right)}{N_{Ti0}}$$

The samples were made from the same stoichiometric green body composition but then hot pressed at different times and temperatures to build the kinetic *α* = *α*(*t*) curves for 1100, 1300, 1400, and 1500 °C. The reaction speed *w = dα*/*dt* dependences were drawn. We assumed that the reaction speed was dependent on the sintering temperature due to Arrhenius’ Equation:

(5) $$w=Ae^{-\frac{E_{a}}{kT}}$$

where *A* is a constant depending on the degree of conversion and *E_a_* is the reaction activation energy. Therefore, plotting *ln*(*w*) on 1/*kT* for a given α we found the activation energy as a slope of the corresponding line.

Finally, two samples were machined with standard metal-working tools (drill, files, hand tap, and die) to create M3 nut and screw.

## 3. Results and Discussion

### 3.1. Densification and Reaction Kinetics

**Figure 1:**
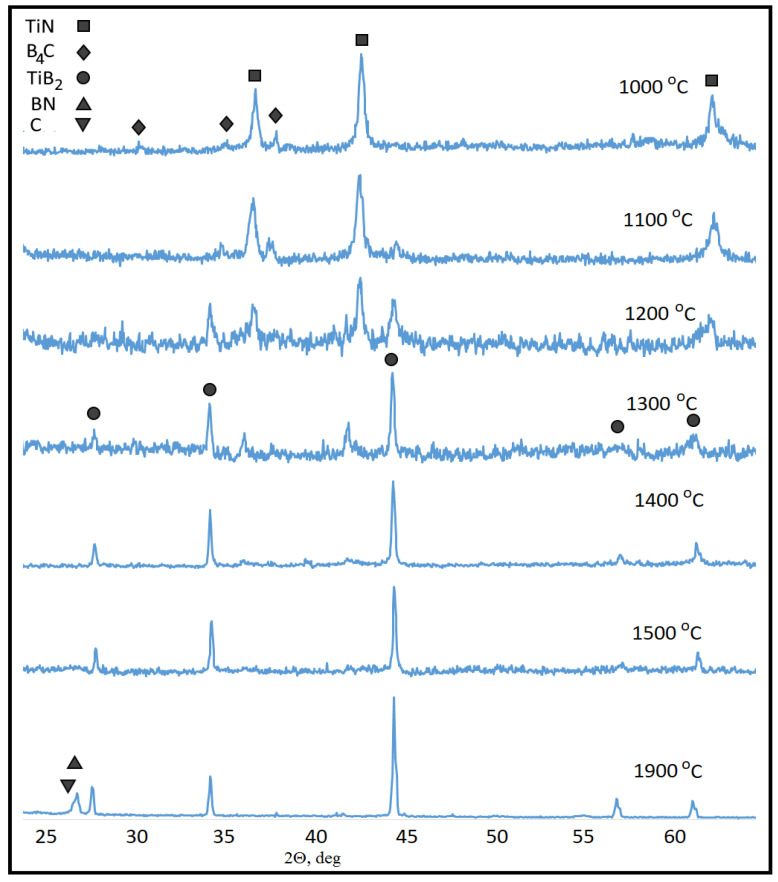
Normalized XRD spectra of 4TiN + 3B_4_C green body after 4-min hot pressing at different temperatures. Peak identification is completed using by Rietveld method.

TiB_2_ formation in TiN-B_4_C was observed already after the hot pressing at 1100 °C (Figure 1). The sintering temperature rise intensified the reaction; therefore, no initial phases can be seen after 4 min of soaking at 1500 °C. The low-intensity halo at 2θ between 26 and 27° is the only very weak signal from carbon and boron nitride in the sample hot-pressed at this temperature.

However, the powder processing at 1900 °C revealed both graphite and h-BN in their crystalline forms (See Figure 1). Therefore, the reaction (2) occurred during the hot pressing of the 4TiN + 3B_4_C green body and resulted in TiB_2_-BN-C HMC formation.

**Figure 2:**
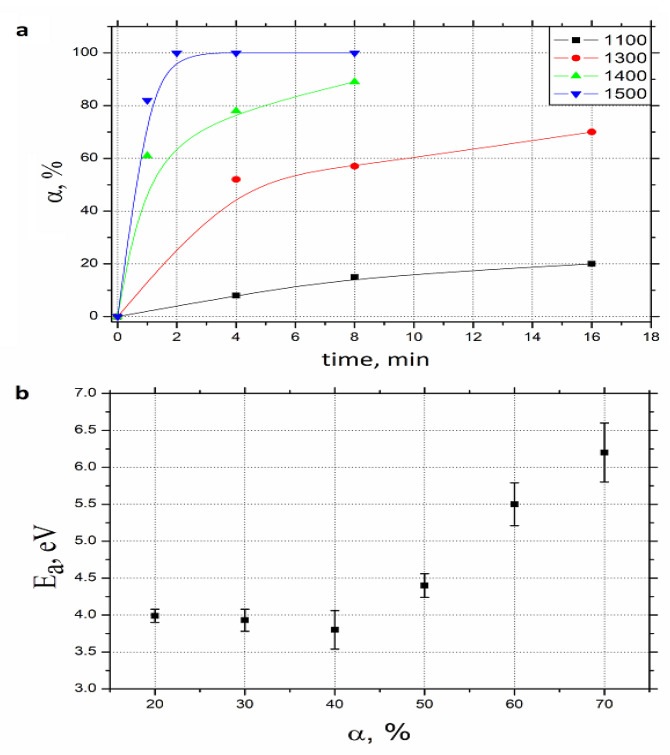
The dependences of the reaction (2) kinetic parameters: the degree of conversion α on time (**a**), and the activation energy (E_a_) on α (**b**).

The phase composition evolution (Figure 2a) shows an essential change between 1300 and 1500 °C with the TiN to TiB_2_ conversion degree not exceeding 70% after 16 min at 1300 °C, and extending up to 100% after 2 min at 1500 °C. The reaction activation energy raises considerably at α > 40% (Figure 2b), thus revealing the change in the main reaction mechanism and its limiting stage. Based on the similarity between TiN and TiC, both having NaCl-type crystalline structure with the lattice parameters of 0.424 nm and 0.433 nm, correspondingly, it is reasonable to assume the similarity of the reaction (2) to the well-investigated one between titanium and boron carbides [33,38]:

2TiC + B_4_C → 2TiB_2_ + 3C (6)

As is shown in [30], the reaction (3) starts at 1100–1200 °C with the boron carbide decomposition [38] and boron diffusion into TiC grains. The process goes along with TiB_2_ nucleation along (111) plains of the carbide crystals. It is possible to assume that the reaction (2) is progressing in the same way. Considering that the value of B_4_C enthalpy does not exceed 63 kJ/moll (~0.1 eV per atom) [39], the material decomposition cannot be the reaction (2) limiting stage (whose activation energy, according to Figure 2b, is at and above 4 eV). The same can be stated about B atoms solid-state diffusion via the product (TiB_2_) phase, whose activation energy is 2.2 eV [40]. This forces us to assume that the reaction is most probably limited with boron atoms transfer through the interphase boundary between B_4_C and TiN. As is shown in [38], solid-state diffusion cannot provide a considerable transformation degree, so the activation energy growth after α = 40% reflects the reaction limiting stage change. According to [30], the intense sublimation of boron atoms from the B_4_C surface begins at 1400 °C, thus the gas phase diffusion became a predominant B to TiN transport mechanism. According to [41], boron sublimation enthalpy approximates 6 eV, which in principle correlates with Figure 2b data and confirms the above-mentioned transport model.

**Figure 3:**
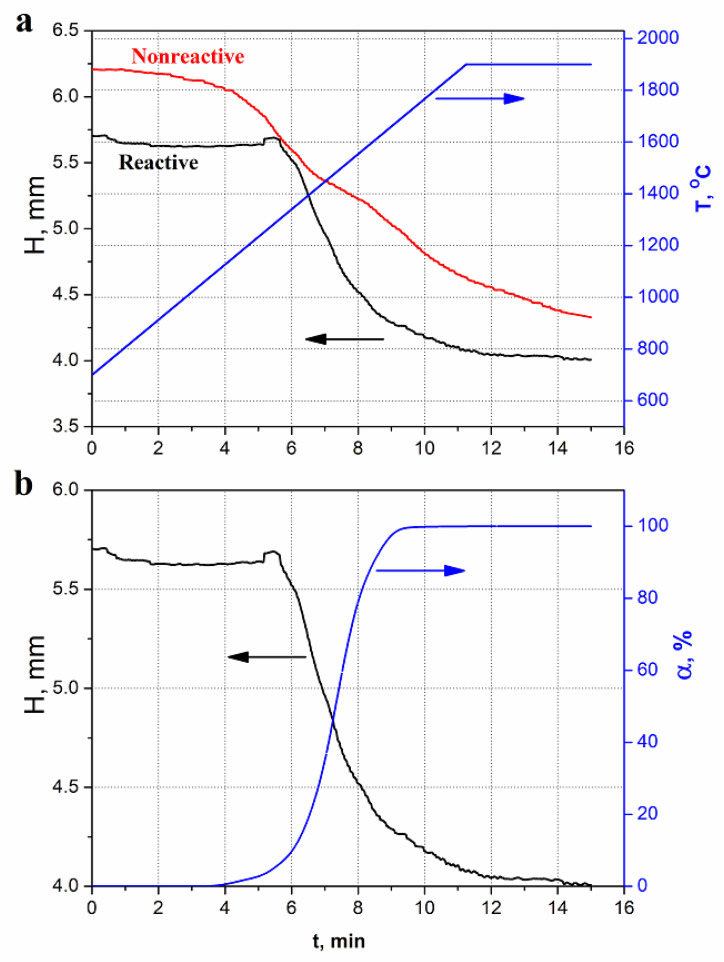
The sample heights (H) (thickness) evolution during hot pressing of the “reactive” (reaction (2) precursor stoichiometry) and “non-reactive” (reaction (2) products) powder mixtures (Table 1) alongside the process temperature (**a**), and the degree of conversion (**b**) for the reactive sample.

Comparison of the “reactive” and “non-reactive” densification kinetics (Figure 3) for two types of green bodies with the same atomic contents of boron, carbon, titanium, and nitrogen, but whose atomic contents are present in different chemical compounds (4TiN + 3B_4_C and 4TiB_2_ + 4BN + 3C) (See Table 1) showed the following:

1. Despite the fact that both mixtures were milled in the same way and the samples contained the same green body mass, at the beginning of the process the non-reactive sample was 10% higher (thicker, e.g., had 10% more volume) than the reactive one. It can be explained on the basis that the bulk density of the non-reactive composition (3.42 g/cm^3^) is 10% lower than that of the reactive one (3.72 g/cm^3^).
2. The non-reactive charge started shrinking (e.g., densifying) after 600 °C while the reactive one remained almost unchanged up to 1250 °C. At 1250 °C, the green body expands and then begins to shrink at 1350 °C. The behavior of the non-reactive powder is most probably mediated by the powder densification helped by the lubrication provided by h-BN and graphite. The reactive sample growth corresponded to the TiB_2_ arrival (the degree of conversion raised from zero, Figure 3b) and could be caused by the reaction (2) dilatometry: the product density being lower than that of the precursors.
3. The non-reactive sample densification proceeds at an almost constant rate over a broad span of time (more than 13 min) and sintering temperature (600 to 1900 °C). Even at 1900 °C and after holding at this temperature for 4 min the densification continues. While the reactive sample densification is almost complete within 5 min and in the interval of 1350–1900 °C.
4. The density of sintered reactive sample is approximately 99% which is 8% higher than that of the non-reactive one.

All listed features are connected to the reaction (2) oddities. Therefore, the in-situ reaction leads to faster ceramic consolidation and denser material.

### 3.2. Atomic Structure Formation

**Figure 4:**
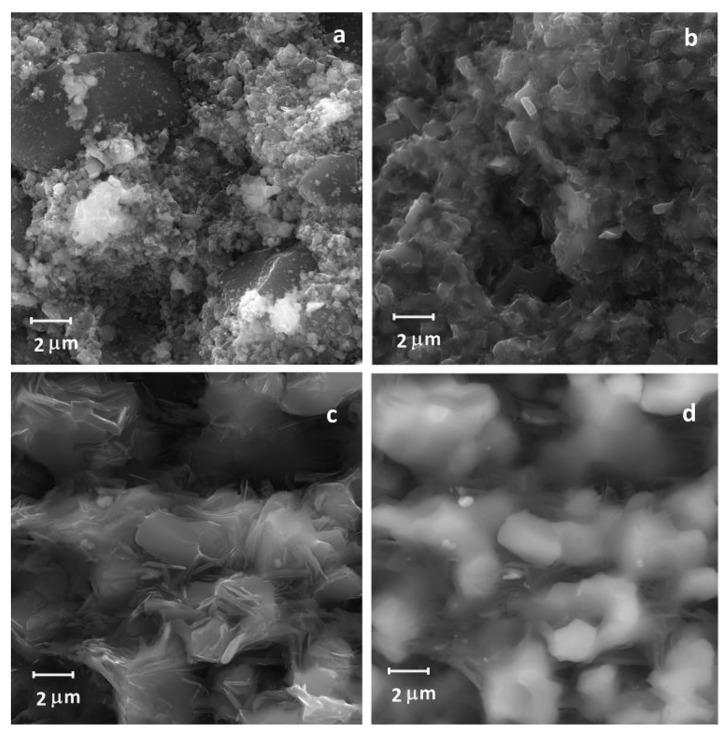
SEM images of the fracture surfaces of TiB_2_-BN-C samples. Hot pressing from TiN-B_4_C powder precursors at: (**a**) 1200 °C (secondary electrons), (**b**) 1650 °C, (secondary electrons), (**c**) 1900 °C (secondary electrons), (**d**) 1900 °C (back scattered electrons).

As shown, the formation of titanium diboride starts at 1100 °C, which is almost two thousand degrees lower than the phase melting point. The TiB_2_ nanocrystals nucleated in such conditions can be observed on the SEM image (Figure 4a) alongside the significantly larger grains which are just the precursor particles. The absence of the rough grains in the sample after hot pressing at 1650 °C (Figure 4b) confirms the completion of TiN to TiB_2_ conversion at the temperature. Nucleated at lower temperatures, TiB_2_ nanoparticles evidently recrystallized into the submicron ones. A hot pressing temperature rise to 1950 °C resulted in further composite structure changes: the diboride grains predictably grew to 2–4 μm, and the new type of plate-like particles appeared on the sample fracture surface (Figure 4c). SEM analysis in back-scattered electrons showed that the plate-like grains contained lighter elements which, considering the XRD results (Figure 1), showed them to be either h-BN or graphite.

**Figure 5:**
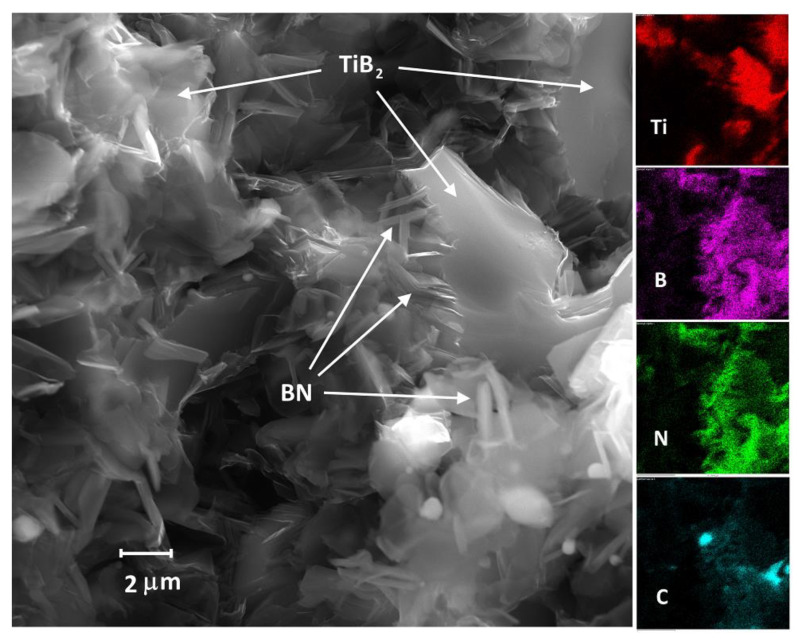
SEM (secondary electrons) and EDS elemental maps of the fractured TiB_2_-BN-C surface when the sample is hot pressed from TiN-B_4_C green body at 1900 °C.

Sample fracture surface investigation with EDS mapping (Figure 5) showed that the plate-like inclusions are boron nitride. Graphite inclusions also seen on the EDS maps, are not in the platelet shape. As was shown in our previous works, TiB_2_-C composites reactively sintered from TiC-B_4_C precursors according to reaction (3), contained two types of carbon particles: the plate-like particles, segregating from TiC after its conversion into TiB_2_, and sponge-like particles, formed with the carbon atoms that remain after boron carbide decomposition [30,33]. Interestingly, the TiN-B_4_C to TiB_2_-BN-C conversion presented here not only repeated the reaction path but also resulted in a similar structure formed via the same reaction mechanism. In which roughly equiaxial 2–4 μm diboride particles nucleated and grew from the parental TiN grains with the displacement of nitrogen by boron and formation of shapeless graphite inclusions after B atoms left B_4_C crystals. BN platelets appear at higher temperatures when the displaced TiN nitrogen reacts with boron.

It should be noted that BN in TiN-B_4_C system formation differs from graphite in TiC-B_4_C segregation. It might be expected that N atoms leaving TiN crystals would form a gaseous N_2_ phase and escape from the material. However, neither XRD nor EDS indicated any free boron, which would have inevitably remained in case of the nitrogen disappearance. As was already stated, boron carbide decomposition was the first stage of the interaction process. Therefore, N atoms leaving TiN particles during their conversion into TiB_2_ nucleus react with boron either on the crystal surface or even before reaching it. This probably explains why the BN structures look to grow (Figure 5) as if from inside of the diboride grains perpendicular to their surfaces.

**Figure 6:**
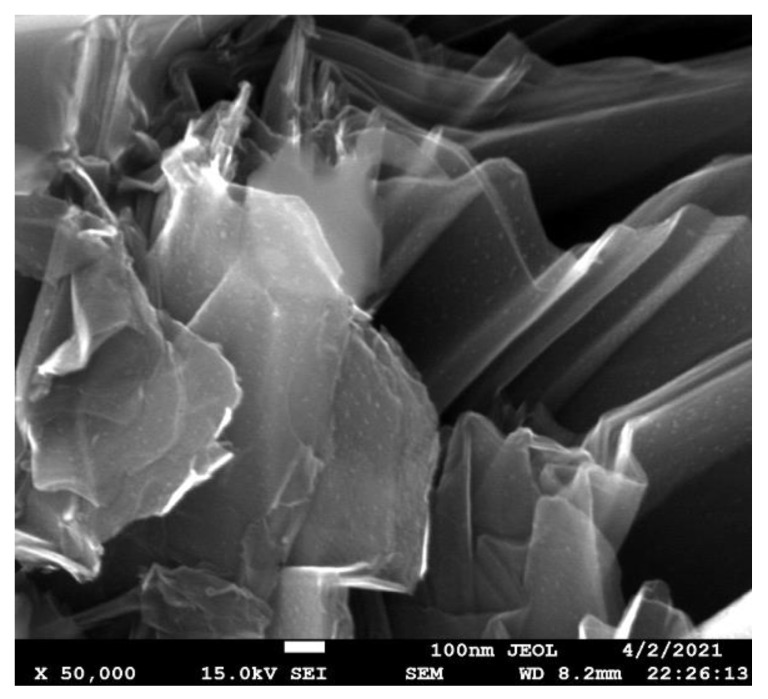
SEM image (secondary electrons) of the BN nanosheets formed near the TiB_2_ grain.

The described growing processes facilitate the formation of BN nanosheets (Figure 6), possibly inside the TiB_2_-based matrix. The nanosheets do not “cover” the diboride grains, allowing the formation of a continuous TiB_2_ frame, which was also confirmed by the fact that the sintered samples were electrically conductive. The possibility of in-situ formation of the h-BN nanostructures is essential considering the difficulties of BN nanosheets manufacturing [42].

**Figure 7:**
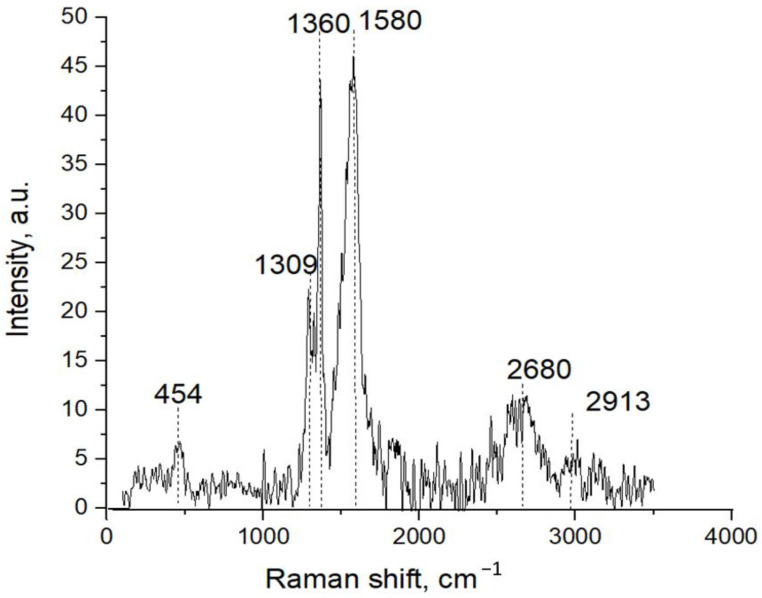
Raman shift spectra of TiB_2_-BN-C ceramics.

Raman spectra of the TiB_2_-BN-C ceramics obtained from the fracture surface are presented in Figure 7. Since the TiB_2_ phase is not Raman active, only *h*-BN and C components can be observed. The characteristic Raman G peak at 1360 cm^−1^ is a fingerprint of *h*-BN, which appears due to in-plane vibrations between B and N atoms [43]. Relatively high peak intensity suggests a multilayer *h*-BN configuration. Other observed features in the Raman spectra at 454, 1309, 1580, and 2680 can be assigned to sp- and sp^2^-bonded carbon. The Raman spectrum of graphite has a main band at 1580 cm^−1^ (D peak) and a small feature at 2687 cm^−1^ (denoted as 2D or G’ peak). Multilayer graphene has additional bands at 1333–1349 and 1604–1618 cm^−1^ [44,45]. These modes are Raman active in defective graphitic materials. In addition, unlike a singlet peak in graphene, the 2D band in multilayer graphene appears as a doublet [46]. The intensity of the second peak in the doublet at approximately 2913 nm^−1^, which is a characteristic of the disorder [47], is very small. Low-frequency peaks observed at 454 cm^−1^ can be attributed to bending modes of sp-coordinated carbon nanostructures [48].

### 3.3. Mechanical Properties

**Figure 8:**
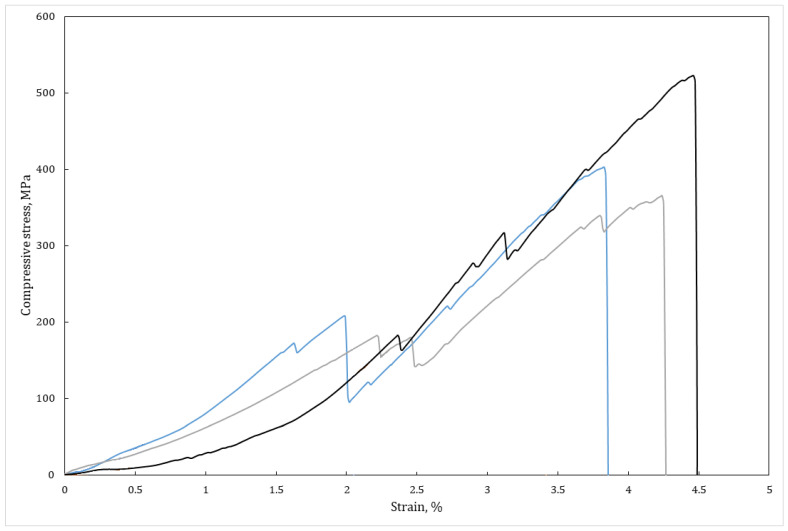
Stress-strain diagram for three reactively pressed TiB_2_-BN-C samples.

Stress-strain curves for three different samples of the TiB_2_-BN-C composite is shown in Figure 8. All investigated samples demonstrated elastic behavior up to approximately 180 MPa. Steps on the curves correspond to the formation of microcracks. Unlike the well-known behavior of other ceramic materials, the crack formation did not result in instant sample destruction. As can be seen from Figure 8, the sintered hetero-modulus composite demonstrates quasiplasticity, when the material deformation is being provided with microcracks formation rather than dislocation movement. Such behavior was explained in [15] with an essential crack deflection on the plate-like soft inclusion. It should be noted, however, that the value of TiB_2_-BN-C composite compressive strength is 2.3 and 1.2 times higher than that of TiC-41C and TiC-33C materials, respectively [15]. Mechanical properties of different soft ceramics used for high-temperature applications are compared in Table 2.

**Figure 9:**
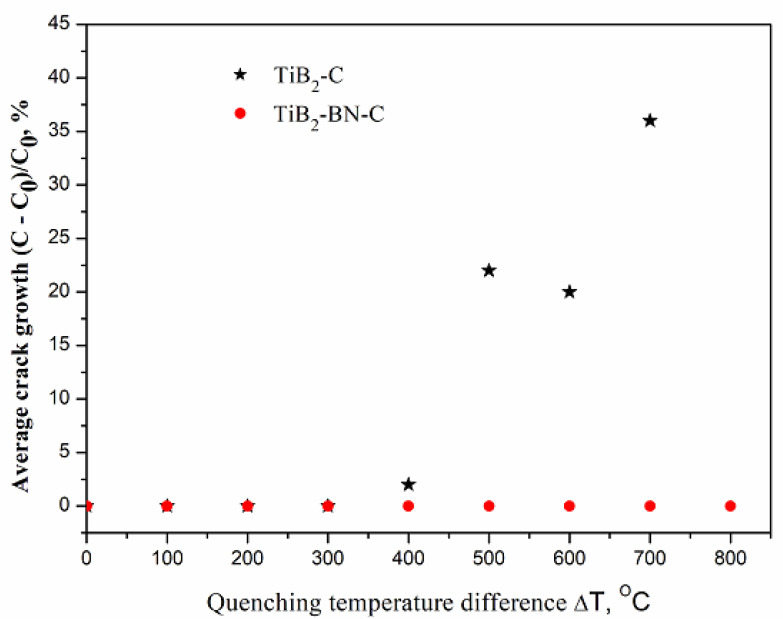
The dependence of the indentation crack growth after quenching from different temperatures of TiB_2_-C (asterisk, [12]) and TiB_2_-BN-C (this work, circles) samples.

As was already shown [12], the indentation crack growth in the TiB_2_-31C sample becomes noticeable when the quenching temperature difference exceeded 400 °C and extended to 36% at ΔT = 700 °C (See Figure 9), which bettered the results [37] substantially. The reactively pressed TiB_2_-33BN-13C material presented even further improvement with the cracks being entirely stable up to quenching temperature differential of 800 °C. The latter coincides with the well-known thermal shock resistance criteria [51,52]:

(7) $$R^{\prime }=\frac{\sigma_{f}\left(1-\nu \right)k}{\alpha E}$$

and

(8) $$R^{\prime \prime }=\frac{K_{1C}\left(1-\nu \right)k}{\alpha E}$$

where $\sigma_{f}$, $K_{1C}$, *ν*, *α*, *k*, and *E* are the fracture strength, toughness, Poisson’s ratio, thermal expansion coefficient, thermal conductivity and Young’s modulus respectively. The investigated reactively pressed ceramic has about 46% of the low Young’s modulus (graphite and h-BN) phases. This allows us to explain the ceramic enhanced, as compared to TiB_2_-31C, thermal shock resistance.

**Figure 10:**
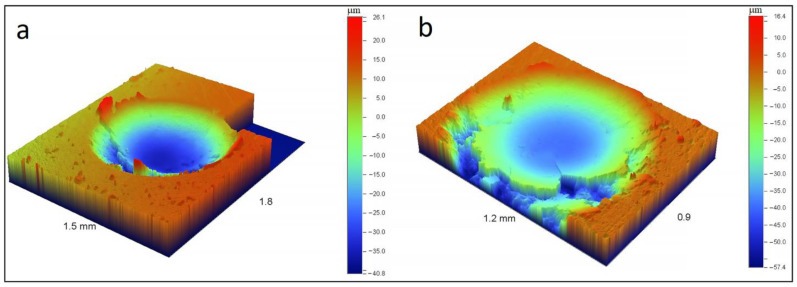
The impact crater for TiB_2_-BN-C (**a**) and TiB_2_-C (**b**) samples.

Comparison of the dynamic impact test results also showed the TiB_2_-BN-C material is less susceptible to mechanical failure compared to the TiB_2_-C counterpart and does not have cracks and damaging zones along the impact cavity edges (Figure 10).

**Figure 11:**
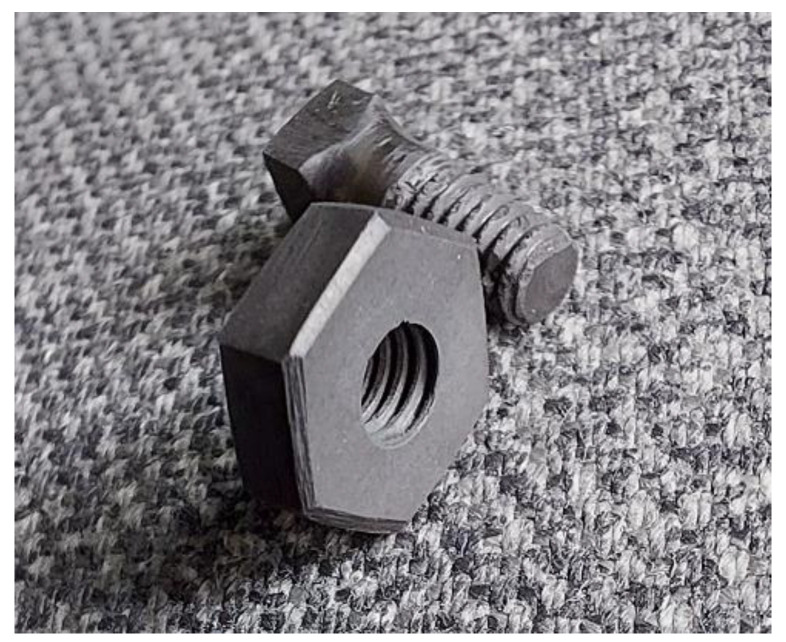
M3 nut and screw produced from the TiB_2_-BN-C ceramic sample.

Finally, as can be seen from Figure 11, the sintered composite is quite machinable. This property presents the possibility of complex shape formation.

## 4. Conclusions

New TiB_2_-BN-C hetero-modulus ceramics with 46% of soft (e.g., low Young’s modulus) phases, h-BN and carbon, were sintered by the in-situ reaction during hot pressing of TiN-B_4_C precursors. TiB_2_ formation was observed already at a temperature around 1100 °C. Reaction activation energy at the beginning of the reaction is at around 4 eV but increases as TiN into TiB_2_ chemical conversion proceeds and reaches values in the region of 6 eV. Nonreactive hot pressing of TiB_2_, BN and carbon precursors proceeds in a wide sintering temperature region from 1100 to 1900 °C but achieves only 92% of the composite theoretical density. Reactive hot pressing of TiN-B_4_C precursors leads to almost full densification (ρ = 99%) of TiB_2_-BN-C hetero-modulus ceramics at 1900 °C. During reactive sintering at temperatures above 1500 °C, multilayer h-BN nanosheets appear. The sintered ceramic exhibits pseudoplastic deformation when microcracks are formed but crack propagation is prevented by the crack tip blunting in the soft inclusions. A high content of low Young’s modulus phases in TiB_2_-33BN-13C ceramic leads to the extremely high thermal shock-resistance when initiated cracks do not grow at least up to quenching temperature differential of 800 °C. The sintered ceramic is easily machinable and can be machined into engineering components subjected to extreme temperature variations.

## Figures and Tables

**Table 1:** The green body compositions.

Title	TiN, wt.%	B_4_C, wt.%	TiB_2_, wt.%	BN, wt.%	C, wt.%
Reactive	60.4	39.6	-	-	-
Nonreactive	-	-	71.1	20.3	8.6

**Table 2:** Some mechanical properties of different soft ceramics for high-temperature applications.

Composition, vol.%	Ref.	Sintering Route	ρ/ρ_th_, %	H_V_, GPa	K_1C_, MPa·m^1/2^	*σ*_flex_, MPa	*σ*_comp_, MPa
TiB_2_-33BN-13C	This work	RHP, 30 MPa, 1900 °C, 4 min	99	1.6	5.3		420
TiB_2_-31C	[12]	RHP, 30MPa, 1800 °C, 8 min	100	4.3	5		
TiC-41C	[15]	HP, 8 MPa, 2700 °C, 30 min	95.7	0.8	5.5	50	180
TiC-33C	[15]	HP, 8 MPa, 2700 °C, 30 min	99.7	1.5	5.5	120	350
TiB_2_-60BN	[49]	HP, 30 MPa, 1850 °C, 90 min	83.9			85	
TiB_2_-50BN	[22]	HP, 30 MPa, 1900 °C, 60 min	71	1.2			
TiB_2_-21TiN-30BN	[50]	SPS, 100 MPa, 1700 °C, 5 min	96	8	<3.7		

## Data Availability

The data presented in this study are available as suplementory material (Raw Data_TiN-B4C.zip).
